# Targeted temperature management after cardiac arrest and fever control with an esophageal cooling device

**DOI:** 10.1186/cc14504

**Published:** 2015-03-16

**Authors:** A Hegazy, D Lapierre, E Althenayan

**Affiliations:** 1 University of Western Ontario, London, ON, Canada

## Introduction

Mild hypothermia and fever control have been shown to improve neurological outcomes post cardiac arrest. Common methods to induce hypothermia include body surface cooling and intravascular cooling; however, a new approach using a catheter placed into the esophagus has recently become available.

## Methods

We report the first three cases of temperature control using an esophageal cooling device (ECD). The ECD was placed orally in a similar fashion to orogastric tubes. Temperature reduction was achieved by connecting the ECD to a commercially available heat exchange unit (Blanketrol II or III).

## Results

The first patient, a 59-year-old male (73 kg), was admitted after successful resuscitation from a protracted out-of hospital cardiac arrest. His initial temperature was 35°C, which is within our current institutional protocol of 34 to 36°C. Several hours after arrival, his temperature slowly increased to 35.8°C despite application of a cooling blanket and ice packs to the groin and axilla. The ECD was inserted and a reduction of temperature to 34.8°C was achieved within 3 hours. The patient expired on day 3. The second patient, a 54-year-old female (95 kg), was admitted after resuscitation from an out-of-hospital PEA arrest. Despite initiating our cooling protocol with surface-cooling blankets and cold intravenous saline, she mounted a fever peaking at 38.3°C shortly after admission. After ECD insertion and confirming the external heat exchanger connection, her temperature gradually dropped to 35.7°C over a period of 4 hours. She subsequently made a favorable neurological recovery and was discharged home at day 23. The third patient, a 47-year-old male patient (86 kg) presented with an ongoing fever secondary to necrotizing pneumonia in the postoperative period after coronary artery bypass grafting. His fever was unresponsive to empiric antibiotic therapy, antipyretics, cooling blankets, and ice packs. ECD insertion resulted in a decrease in temperature from 39.5°C to 36.5°C in less than 5 hours. The patient eventually made a full recovery and was discharged home after 59 days. In all three patients, placement of the device occurred in less than 3 minutes and ease of use was reported as excellent by nursing staff and physicians.

## Conclusion

The ECD is a novel technology that can be used for temperature management post cardiac arrest and for fever control in critically ill patients. Despite patients mounting a febrile response, temperature control was achieved and maintained successfully. The device was reported as being easy to use, by both physicians and nurses.

